# Topical versus oral metronidazole for post‐haemorrhoidectomy pain: A systematic review and meta‐analysis of randomized controlled trials

**DOI:** 10.1111/codi.70321

**Published:** 2025-11-30

**Authors:** Bernardo Fontel Pompeu, Giulia Luiza Garcia, Gabriel Leal Barone, Isabela Oliveira Rosa, Lucas Soares de Souza Pinto Guedes, Lucas Monteiro Delgado, Claudia Theis, Leonardo de Melo Del Grande, Sergio Mazzola Poli de Figueiredo, Fernanda Bellotti Formiga

**Affiliations:** ^1^ Department of Colorectal Surgery Heliopolis Hospital São Paulo Brazil; ^2^ University of São Caetano do Sul São Caetano do Sul São Paulo Brazil; ^3^ Federal University of Minas Gerais Belo Horizonte Brazil; ^4^ Department of Surgery University of North Carolina Chapel Hill North Carolina USA; ^5^ Federal University of São Paulo São Paulo Brazil; ^6^ Department of Colorectal Surgery Hospital of Santa Casa de Misericordia São Paulo Brazil

**Keywords:** haemorrhoidectomy, oral metronidazole, post‐operative pain, randomized controlled trial, topical metronidazole

## Abstract

**Background:**

Excisional haemorrhoidectomy is often associated with intense post‐operative pain and delayed recovery. Bacterial colonization may contribute to this discomfort, and metronidazole has been proposed as an analgesic adjunct. This study compared the analgesic efficacy of topical metronidazole with oral metronidazole.

**Methods:**

A systematic search was conducted in PubMed, Scopus, and the Cochrane Central Register through May 2025. Randomized controlled trials comparing topical metronidazole with oral metronidazole for post‐operative pain after haemorrhoidectomy were included. Visual analogue scale (VAS) scores on post‐operative days 1, 3, and 7 were pooled. Mean differences (MDs) with 95% confidence intervals (CIs) were calculated using a random‐effects model. Heterogeneity was assessed with the *I* ^2^ statistic. Risk of bias (RoB 2) and certainty of evidence (GRADE) were evaluated.

**Results:**

Four RCTs involving 439 patients (218 topical, 221 oral) met inclusion criteria. No significant differences were found between topical and oral metronidazole at day 1 (MD –0.1; 95% CI –0.3 to 0.2; *I* ^2^ = 25%), day 3 (MD –0.4; 95% CI –1.3 to 0.6; *I* ^2^ = 94%), or day 7 (MD –0.2; 95% CI –1.0 to 0.5; *I* ^2^ = 90%).

**Conclusion:**

Topical and oral metronidazole showed similar short‐term analgesic efficacy after haemorrhoidectomy.

**What does this paper add to the literature?**

This study provides the first quantitative head‐to‐head comparison between topical and oral metronidazole after haemorrhoidectomy. By synthesizing all available randomized trials, it demonstrates equivalent short‐term analgesic efficacy between routes, clarifies uncertainty in clinical practice, and refines post‐operative pain management by addressing a previously unanswered question in the literature.

## INTRODUCTION

Haemorrhoids are one of the most prevalent anorectal conditions, affecting up to 35% of the general population. [1, 2, 3]. Excisional procedures, although effective, are frequently associated with substantial post‐operative pain, necessitating a multimodal pain management strategy aimed at minimizing discomfort while reducing opioid use, thereby lowering the risk of dependence and contributing to efforts against the opioid crisis [4, 5, 6, 7].

The primary mechanisms underlying post‐operative pain after haemorrhoidectomy include an intense inflammatory response to tissue injury, spasm of the internal anal sphincter, and secondary bacterial colonization of the surgical site [8, 9, 10, 11]. These factors contribute to nociceptive sensitization and delayed recovery. To address these mechanisms, a variety of analgesic interventions are employed, including NSAIDs, corticosteroids, pudendal nerve block, liposomal bupivacaine, lidocaine, glyceryl trinitrate, calcium channel blockers (e.g., diltiazem), botulinum toxin, sucralfate, metronidazole, and gabapentin [4, 5, 8, 9, 10, 11]. Recent studies have suggested that metronidazole, administered either topically or orally, can significantly reduce post‐operative pain [8, 9, 10, 11, 12, 13, 14, 15].

Two previous meta‐analyses have evaluated metronidazole versus placebo for pain control after excisional haemorrhoidectomy [16, 17]. One of them demonstrated that both topical and oral administration provide effective analgesia [16]. In addition, a recent systematic review by Eberspacher et al. provided a narrative and scoping synthesis of topical metronidazole use, including comparisons with both placebo and oral formulations [18]. However, that review did not perform a quantitative head‐to‐head analysis between topical and oral administration. The present study aimed to address this gap by comparing these two forms exclusively in randomized controlled trials.

## METHODS

This systematic review was conducted in accordance with the PRISMA (Preferred Reporting Items for Systematic Reviews and Meta‐Analyses) guidelines, as outlined in Table S1 [19]. The protocol was prospectively registered in the International Prospective Register of Systematic Reviews (PROSPERO; registration number CRD420251038449) [20]. As the analysis was based solely on data from previously published studies, ethical approval was not required. All sources were appropriately cited, and standardized terminology was applied to ensure methodological consistency. These procedures align with the Committee on Publication Ethics (COPE) recommendations, which allow limited text reuse when necessary to ensure clarity, transparency, and reproducibility [21].

### Systematic search process

We conducted a comprehensive literature search using PubMed, the Cochrane Central Register of Clinical Trials, and Scopus to identify studies published up to May 2025. The PubMed search strategy was: (“Hemorrhoidectomy”[Mesh] OR “Hemorrhoidectomy” OR “haemorrhoidectomy” OR “Hemorrhoidal surgery” OR “Hemorrhoids surgery” OR “hemorrhoid” OR “haemorrhoid” OR “hemorrhoidectomy procedure”) AND (“postoperative pain” OR “post haemorrhoidectomy pain” OR “postsurgical pain” OR “post‐surgical pain” OR “post‐operative pain” OR “Pain, Postoperative”[Mesh] OR “Pain, Postoperative” OR “Surgical site pain” OR “Pain following surgery” OR “post hemorrhoidectomy pain” OR “mean pain score” OR “VAS score” OR “Visual analogue scale” OR “pain score” OR “pain relief” OR “analgesia” OR “analgesic effect”) AND (“Metronidazole”[Mesh] OR “Metronidazole” OR “Flagyl” OR “topical metronidazole” OR “Metronidazole gel” OR “nitroimidazole” OR “antimicrobial therapy” OR “Administration, Topical”[Mesh] OR “Administration, Topical”).

The original Scopus and Cochrane search strategy was: (“Hemorrhoidectomy” OR “haemorrhoidectomy” OR “Hemorrhoidal surgery” OR “Haemorrhoids surgery” OR “hemorrhoid” OR “haemorrhoid” OR “hemorrhoidectomy procedure”) AND (“postoperative pain” OR “post haemorrhoidectomy pain” OR “postsurgical pain” OR “post‐surgical pain” OR “post‐operative pain” OR “Pain, Postoperative” OR “Surgical site pain” OR “Pain following surgery” OR “post hemorrhoidectomy pain” OR “mean pain score” OR “VAS score” OR “Visual analogue scale” OR “pain score” OR “pain relief” OR “analgesia” OR “analgesic effect”) AND (“Metronidazole” OR “Flagyl” OR “topical metronidazole” OR “Metronidazole gel” OR “nitroimidazole” OR “antimicrobial therapy” OR “Administration, Topical”).

### Study selection criteria

We included randomized controlled trials (RCTs) that compared post‐operative topical versus oral metronidazole in patients undergoing excisional haemorrhoidectomy, regardless of haemorrhoid grade. No language restrictions were applied during the search process.

Exclusion criteria were as follows: (1) studies not involving haemorrhoidectomy procedures; (2) studies addressing other benign anorectal conditions; (3) studies exclusively evaluating oral or topical metronidazole versus placebo; (4) studies lacking a comparative control group; (5) publications in non‐eligible formats, such as single‐arm trials, case reports, conference abstracts, meta‐analyses, narrative reviews, or animal studies; and (6) studies with overlapping patient populations, in which the most recent or most comprehensive data set was prioritized.

### Data extraction and endpoints

Two independent reviewers (G.L.B. and G.L.G.) screened all retrieved articles for eligibility and extracted data from studies that met the inclusion criteria. Discrepancies were resolved by consensus, and when necessary, adjudication was provided by a third reviewer (B.F.P.). Only outcomes reported with comparable measures in at least three studies were quantitatively synthesized. Accordingly, the primary analysis compared topical and oral metronidazole in terms of post‐operative pain, as assessed by the visual analogue scale (VAS), on post‐operative days 1, 3, and 7.

### Quality assessment

The risk of bias for each randomized controlled trial (RCT) was independently evaluated by two reviewers (G.L.B. and G.L.G.) using the Revised Cochrane Risk of Bias Tool (RoB 2) [22]. Studies were classified as having low risk, high risk, or raising some concerns across five key domains: randomization process, adherence to intended interventions, completeness of outcome data, accuracy of outcome measurement, and selective reporting of results. Any discrepancies between reviewers were resolved through discussion, with a third or fourth reviewer (F.B.F. or B.F.P.) acting as arbiter when necessary. The certainty of evidence for each endpoint was assessed using the GRADE approach (Grading of Recommendations Assessment, Development, and Evaluation), with confidence rated as very low, low, moderate, or high [23]. Assessment of publication bias, including visual inspection of funnel plot symmetry and Egger's regression test, was not performed due to the small number of included studies, as these methods are recommended only when at least nine studies are available for a given outcome [24, 25, 26].

### Statistical analysis

Continuous outcomes were analysed using mean differences (MDs) with 95% CIs. All pooled estimates were generated using a random‐effects model according to the DerSimonian and Laird method [27]. Statistical significance was defined as a p‐value <0.05. Between‐study heterogeneity was examined using Cochran's Q test and quantified with the *I* ^2^ statistic, with *I* ^2^ values >25% and p‐values <0.10 indicating substantial heterogeneity. All analyses were performed using R software, version 4.4.1, using the ‘meta’ and ‘metafor’ packages.

### Sensitivity analysis

For outcomes with substantial heterogeneity (*I* ^2^ > 25%), two complementary sensitivity analyses were performed. First, Baujat plots were generated to identify the studies that contributed most to both the overall effect size and the heterogeneity observed across trials [27]. Second, a leave‐one‐out approach was implemented, systematically omitting one study at a time to assess the robustness of the results and the influence of each trial.

## RESULTS

### Study inclusion and baseline characteristics

**FIGURE 1:**
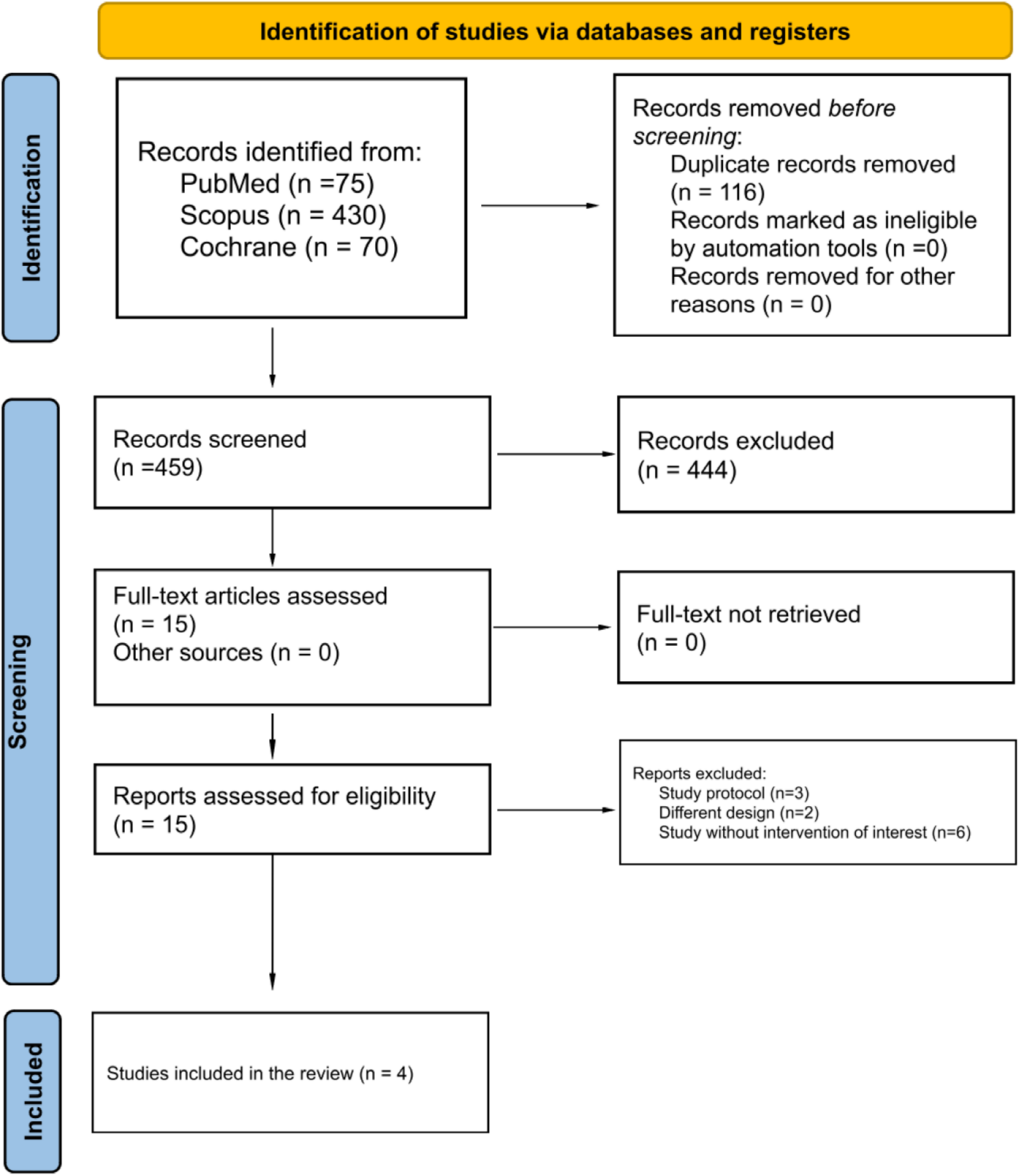
PRISMA flow diagram of study screening and selection.

As shown in Figure 1, the systematic search initially identified 575 records. After excluding 116 duplicates and 459 studies based on title and abstract screening, 15 full‐text articles were assessed for eligibility. Ultimately, four randomized controlled trials (RCTs) comparing topical and oral metronidazole met the inclusion criteria and were incorporated into the final analysis [8, 9, 10, 11]. Collectively, these studies enrolled 439 patients, with 218 (49.7%) allocated to the topical group and 221 (50.3%) to the oral group [8, 9, 10, 11]. All included trials administered topical and oral metronidazole (10% ointment or 400 mg tablets) for 5 to 7 days, although two studies did not specify dosing frequency [9, 10]. The follow‐up duration ranged from 5 to 14 days across the studies. The overall proportion of male participants was 57.2% (*n* = 251), with a mean age of 41.9 ± 5.4 years. Regarding disease severity, among the studies reporting haemorrhoid grade (*n* = 3), grade III accounted for 63.1% and grade IV for 36.9% of cases. In terms of surgical technique, Milligan–Morgan haemorrhoidectomy was performed in 77.7% of patients, while the Ferguson approach was used in 22.3%, with one study reporting a mixed distribution between the two techniques [8, 9, 10, 11].

Post‐operative analgesia protocols varied across studies, but all included nonsteroidal anti‐inflammatory drugs (NSAIDs) as the mainstay, with opioids administered as rescue medication [8, 9, 10, 11]. Diclofenac was the most reported NSAID, administered intramuscularly or orally, while flurbiprofen was used in only one trial [11]. Opioid use, when reported, is typically reserved for breakthrough pain, with tramadol mentioned explicitly in one trial. Two studies did not provide detailed information on opioid regimens [9, 10]. Unfortunately, other post‐operative pain‐related outcomes could not be analysed quantitatively due to inconsistent or missing data. Likewise, additional secondary outcomes, such as wound healing, infection, and other complications, were not comparable across trials and were therefore excluded from the pooled analysis.

Among the included RCTs, only Xia et al. implemented a double‐blind design, using identical placebo capsules and ointments to ensure masking of both participants and investigators [11]. The remaining three studies were *open‐label*, as the route of administration (topical versus oral) precluded effective blinding [8, 9, 10]. All trials assessed post‐operative pain using the visual analogue scale (VAS, 0–10 cm), with evaluations performed between the 1st and 14th post‐operative days [8, 9, 10, 11]. Pain was measured at rest and/or during defaecation, and in all cases, assessment relied on self‐reported patient scores [8, 9, 10, 11]. None of the studies described blinded outcome evaluators. Therefore, outcome measurement was considered subjective across the included trials [8, 9, 10, 11]. A detailed overview of the remaining study characteristics is presented in Tables 1, 2, and S2.

**TABLE 1:** Basic characteristics of the included randomized controlled trials.

Author	Country	Topical/Oral	Design	Sex (male)	Age (years)	BMI	3° degree haemorrhoids	4° degree haemorrhoids	Follow‐up^a^
				*n* (%)	Mean ± SD	Mean ± SD	*n* (%)	*n* (%)	
				Topical/Oral	Topical/Oral	(kg/m^2^)	Topical/Oral	Topical/Oral	
Abbas ST 2020	Pakistan	83/83	RCT	55 (66.27)/56 (67.47)	44 ± 10.45/43 ± 10.84	NA	66 (79.5)/68 (81.9)	17 (20.5)/15 (18.1)	7 days
Neogi P 2018	India	20/20	RCT	20 (100)/20 (100)	NA	NA	NA	NA	7 Days
Razzaq S 2020	Pakistan	60/60	RCT	76 (63.33)	38.74 ± 11.42	24.52 ± 2.86	53 (44.16)	67 (55.83)	5 Days
Xia W 2022	New Zealand	55/58	RCT	25 (41.7)/22 (36.7)	45.5 (18)/45.5 (18)**	27.1 (9.2)/26.2 (8)**	34 (56.67)/31 (51.7)	26 (43.3)/29 (48.3)	14 Days

Abbreviations: NA, not available; RCT, randomized controlled trial.

^a^ Follow‐up = last post‐operative day of pain assessment; not mean or median.

** Median (IQR).

**TABLE 2:** Surgical techniques and metronidazole regimens in the included randomized controlled trials.

Author	Milligan–Morgan	Ferguson surgical approach	Metronidazole dose and administration	Metronidazole posology	Post‐operative analgesia/anaesthesia protocol
	*n* (%)	*n* (%)	Topical × Oral	Topical × Oral	
	Topical × Oral	Topical × Oral			
Abbas 2020	83 (100)/83 (100)	0/0	Metronidazole 10% ointment/Metronidazole 400 mg tablets	6 h post‐operatively, then every 8 h for 7 days/every 8 h for 7 days	Diclofenac 75 mg IM 12/12 h for 2 days; opioids if required
Neogi 2018	20 (100)/20 (100)	0/0	Metronidazole 10% ointment/Metronidazole 400 mg tablets	Twice daily for 7 days/Three times daily for 7 days	Diclofenac + tramadol as needed
Razzaq 2020	60 (100)/60 (100)	0/0	Metronidazole 10% ointment/Metronidazole 400 mg tablets	Twice daily for 5 days/Three times daily for 5 days	Diclofenac sodium 75 mg IM; opioids if severe pain
Xia 2022	14 (23.3)/8 (13.3)	16 (26.7)/23 (38.3)	Metronidazole 10% ointment + placebo tablets/400 mg tablets + placebo ointment	Three times daily for 7 days/Three times daily for 7 days	1 g paracetamol 4× daily; 400 mg ibuprofen 3× daily; lactulose; psyllium; 50–100 mg tramadol as needed (flurbiprofen for intense pain)

### Pooled analyses of the included studies

#### Post‐operative pain

**FIGURE 2:**
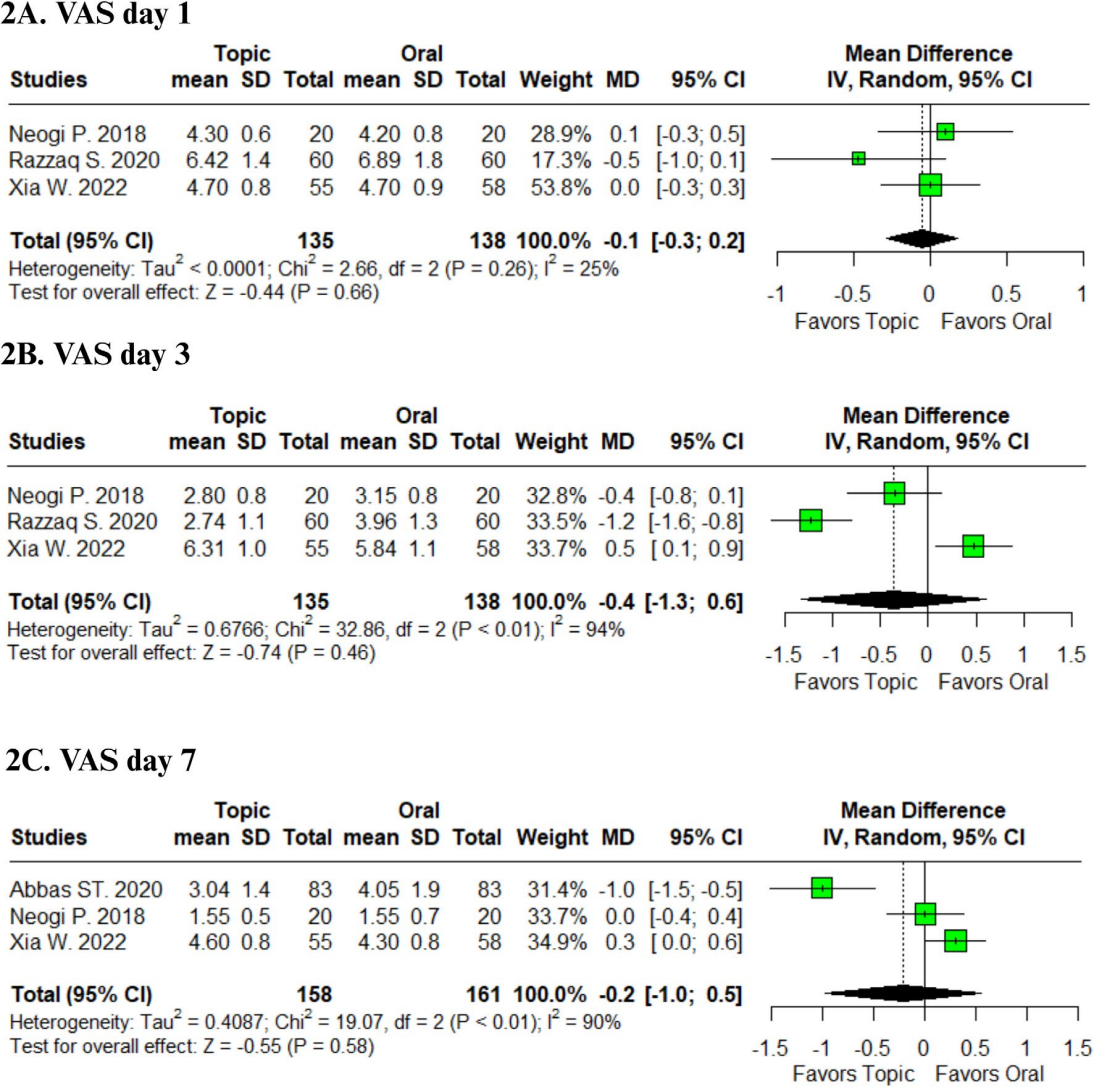
Forest plots comparing topical and oral metronidazole after conventional haemorrhoidectomy: (A) VAS score day 1, (B) VAS score day 3, (C) VAS score day 7.

When comparing topical versus oral metronidazole, no significant differences were detected for VAS at Day 1 (MD –0.10; 95% CI –0.30 to 0.20; *I* ^2^ = 25%, Figure 2A), Day 3 (MD –0.40; 95% CI –1.30 to 0.60; *I* ^2^ = 94%, Figure 2B), and Day 7 (MD –0.20; 95% CI –1.00 to 0.50; *I* ^2^ = 90%, Figure 2C). These outcomes demonstrated low heterogeneity for Day 1, while Days 3 and 7 showed high heterogeneity [8, 9, 10, 11].

### Sensitivity analyses

Baujat plot analyses were employed to identify the studies contributing most to heterogeneity across outcomes. For VAS at post‐operative day 1, Razzaq et al. were identified as the main contributor, although exclusion of their study did not alter pooled estimates (Figures S1 and S2) [8, 9, 10, 11]. For VAS at post‐operative day 3, both Xia and Razzaq contributed most to the heterogeneity, yet their removal did not meaningfully impact the results (Figures S3 and S4) [10, 11]. Finally, regarding VAS day 7, Abbas and Xia emerged as the primary drivers of heterogeneity, but their exclusion did not affect the overall findings (Figures S5 and S6) [8, 11].

### Quality assessment and certainty

**FIGURE 3:**
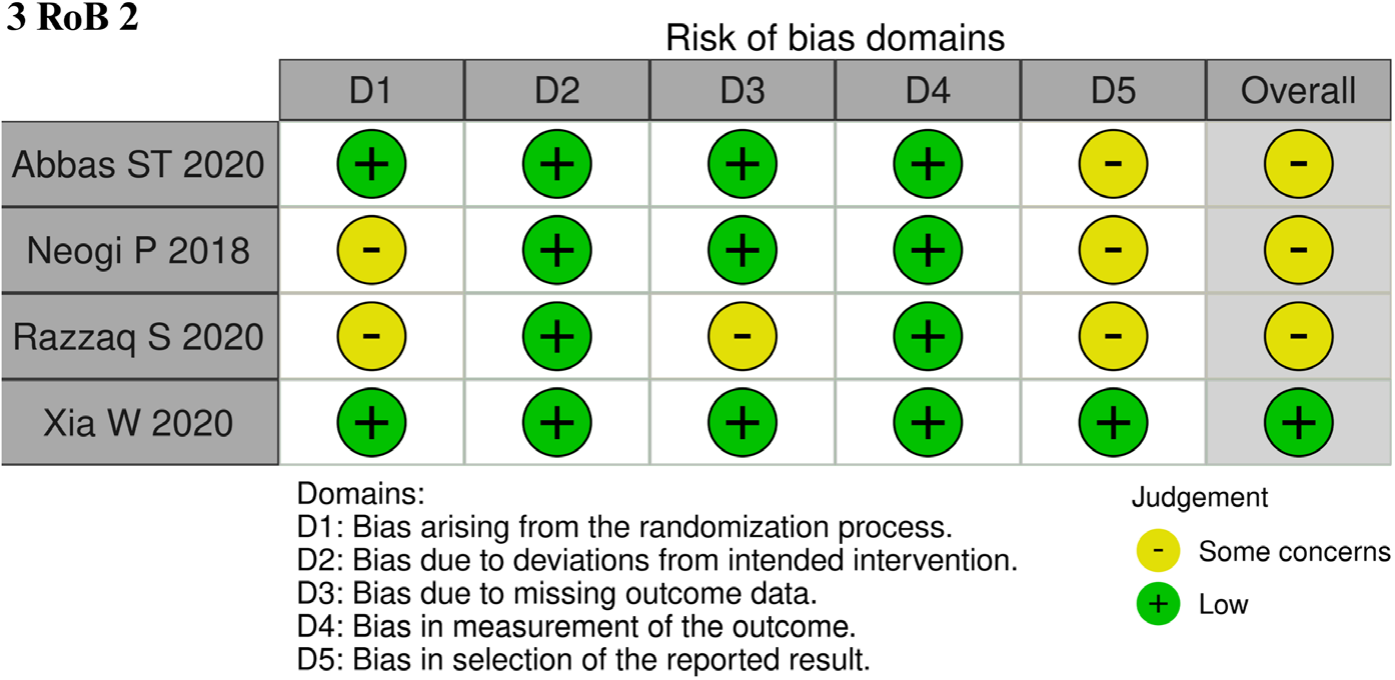
Critical appraisal of RCTs according to the Cochrane Collaboration's tool for assessing risk of bias (Rob 2).

An overview of the study‐specific risk of bias assessments conducted in this meta‐analysis is provided in Figure 3 and Table S3. All four included RCTs were evaluated using the RoB 2 tool. Among them, only Xia et al. were judged to have a low overall risk of bias across all domains [11]. Two trials (Neogi and Razzaq) presented some concerns in domain 1 (randomization process), mainly due to insufficient details on allocation sequence generation and concealment, which raised uncertainty regarding baseline comparability [9, 10]. Razzaq also raised concerns in domain 3 (missing outcome data), as the handling of loss to follow‐up was not adequately described [10]. Furthermore, three studies presented some concerns in domain 5 (selection and reporting of results) [8, 9, 10], primarily due to the absence of prospective trial registration or a pre‐specified analysis plan, which limits confirmation that all intended outcomes were reported without selective reporting bias. Overall, the risk of bias was rated as “some concerns” in three trials and “low” in one [8, 11].

The GRADE evaluation revealed that for VAS on Day 1, the certainty of evidence was rated as moderate, downgraded by one level due to serious risk of bias, as most trials presented some concerns and only one had low risk. For VAS on Day 3 and Day 7, the certainty of evidence was rated as very low, downgraded by three levels due to serious risk of bias, serious inconsistency related to substantial heterogeneity (*I* ^2^ = 94% and *I* ^2^ = 90%, respectively), and serious imprecision with wide confidence intervals crossing the line of no effect. Full details of the GRADE assessments are available in Table S4.

## DISCUSSION

In this systematic review and meta‐analysis of four randomized controlled trials including 439 patients undergoing excisional haemorrhoidectomy, the comparison was made between topical and oral metronidazole. No significant differences were observed in pain scores at post‐operative days 1, 3, and 7 between topical and oral administration.

Our study directly addresses a key research gap highlighted in previous studies, namely the lack of a head‐to‐head comparison between topical and oral metronidazole for post‐operative pain control after haemorrhoidectomy [8, 9, 10, 11, 12, 13, 14, 15]. While earlier studies and reviews focused on the analgesic effect of metronidazole versus placebo, none have systematically evaluated whether one administration route offers superior pain relief [16, 17]. By pooling only randomized controlled trials, this meta‐analysis provides the first high‐level evidence that topical and oral formulations of metronidazole achieve comparable short‐term analgesic outcomes following haemorrhoidectomy. This finding carries important clinical relevance since topical administration could, in theory, reduce systemic drug exposure, potentially minimizing adverse events and limiting the development of antimicrobial resistance. However, none of the included trials directly assessed these outcomes, underscoring the need for further research.

Excisional haemorrhoidectomy remains the only definitive surgical cure for grades III and IV haemorrhoids, or for those with complications [28]. Post‐operative pain is one of its main challenges, affecting up to 40% of patients [29]. Large‐scale data from over 50,000 patients in Germany identified haemorrhoidectomy as one of the procedures with the highest post‐operative pain scores, often exceeding those of major abdominal surgeries, due to intense nociceptive stimulation and limited use of regional analgesia [29]. These findings highlight the need for procedure‐specific pain management protocols and close post‐operative monitoring, even in so‐called minor surgeries.

The pain‐relieving effect of metronidazole likely results from multiple mechanisms [8, 9, 10, 11, 12, 13, 14, 15]. In addition to its antimicrobial role, it may attenuate the release of inflammatory mediators triggered by surgical injury, thereby alleviating muscle spasm and tissue swelling [30, 31]. By controlling local bacterial colonization, it can also minimize the risk of microabscess formation, which contributes to post‐operative discomfort [5]. Furthermore, metronidazole exhibits anti‐inflammatory and antioxidant properties, which limit the production of reactive oxygen species and pro‐inflammatory cytokines [30, 31]. This combination of effects may underlie its analgesic benefits, which are not consistently reproduced with other antibiotic agents [16].

The recently published TAPH trial further supports the analgesic rationale for topical metronidazole after haemorrhoidectomy [32]. This RCT compared metronidazole alone with combinations including diltiazem and lidocaine, finding no additional benefit from adjunctive agents [32]. These findings reinforce the consistent efficacy of topical metronidazole as a standalone option. Although the study did not include an oral comparator, its results align conceptually with our analysis, confirming metronidazole's central role in post‐operative pain control following excisional haemorrhoidectomy [32].

Nicholson et al. conducted a prospective, randomized trial evaluating 10% topical metronidazole after Harmonic Scalpel® haemorrhoidectomy and reported significantly lower pain scores at days 7 and 14, less post‐operative oedema, and improved overall wound healing compared with placebo [5]. Similarly, Ala et al. performed a double‐blind randomized study in 47 patients and found that topical metronidazole significantly decreased both resting and post‐defaecation pain up to day 14, with a reduced need for additional analgesics on days 2 and 7 [4]. In line with these findings, our results confirm that topical metronidazole offers a safe, well‐tolerated, and effective adjunct for enhancing post‐operative pain control, particularly when compared with placebo.

Given the similar efficacy, the choice between topical and oral metronidazole should rely on secondary factors such as patient preference, tolerability, safety profile, cost, and ease of administration. Topical formulations may be advantageous in reducing systemic exposure and gastrointestinal adverse effects, whereas oral therapy could offer greater convenience and compliance. Overall, the available evidence indicates that both formulations are effective adjuncts for post‐operative pain management following haemorrhoidectomy, and the selection should be guided by patient‐ and context‐specific considerations rather than efficacy alone.

It is noteworthy that considerable heterogeneity was observed in post‐operative pain scores, particularly on days 3 and 7. This variability likely reflects differences among studies in surgical techniques (Milligan–Morgan versus Ferguson), timing of pain assessment, metronidazole dosing and formulation, and perioperative analgesic protocols. Therefore, these findings should be interpreted with caution, as the limited number of included trials and small sample size may have contributed to the observed variability.

This meta‐analysis has some limitations that should be considered when interpreting the findings. First, only four randomized controlled trials with a total of 439 patients undergoing haemorrhoidectomy were included, comparing oral versus topical metronidazole. The relatively small sample sizes limit the statistical power and generalizability of the results. Second, substantial heterogeneity was observed for several outcomes, such as post‐operative pain scores at days 3 and 7, which may be attributed to differences in surgical techniques, analgesic regimens, metronidazole dosing, and follow‐up protocols. Third, the risk‐of‐bias assessment revealed that most trials lacked prospective registration and pre‐specified analysis plans, increasing the possibility of selective outcome reporting. Fourth, variations in surgical approaches (Milligan–Morgan vs. Ferguson techniques) and perioperative pain management strategies may have influenced the analgesic outcomes. Additionally, other post‐operative pain‐related and secondary outcomes could not be analysed quantitatively because of incomplete or inconsistent reporting across trials, which limited the comprehensiveness of the pooled analysis. Finally, although metronidazole consistently demonstrated analgesic benefits compared with placebo, the overall certainty of the current evidence remains low. Most available studies were small, single‐centre trials with methodological heterogeneity and short follow‐up, underscoring the need for larger, well‐designed RCTs to confirm these findings and better define the clinical role of metronidazole in post‐haemorrhoidectomy pain management.

## CONCLUSION

In this systematic review and meta‐analysis of four randomized controlled trials including 439 patients undergoing excisional haemorrhoidectomy, topical and oral metronidazole were directly compared. No significant differences were observed in post‐operative pain scores at days 1, 3, and 7, indicating that both routes of administration provide comparable analgesic benefits in the early recovery period.

## AUTHOR CONTRIBUTIONS

**Bernardo Fontel Pompeu:** Conceptualization; investigation; funding acquisition; writing – original draft; methodology; validation; writing – review and editing; visualization; software; formal analysis; project administration; resources; supervision; data curation. **Giulia Luiza Garcia:** Investigation; writing – original draft; methodology; validation; writing – review and editing; software; visualization; formal analysis; project administration; data curation; resources. **Gabriel Leal Barone:** Conceptualization; investigation; writing – original draft; methodology; validation; visualization; writing – review and editing; software; formal analysis; resources; data curation. **Isabela Oliveira Rosa:** Investigation; writing – original draft; methodology; validation; visualization; writing – review and editing; formal analysis; software; data curation; resources. **Lucas Soares de Souza Pinto Guedes:** Data curation; resources; formal analysis; software; methodology; validation; visualization; writing – review and editing; investigation; writing – original draft. **Lucas Monteiro Delgado:** Investigation; writing – original draft; methodology; validation; visualization; writing – review and editing; software; formal analysis; data curation; resources. **Claudia Theis:** Conceptualization; writing – original draft; investigation; methodology; validation; visualization; writing – review and editing; software; project administration; supervision; resources; data curation. **Leonardo de Melo Del Grande:** Conceptualization; investigation; funding acquisition; writing – original draft; methodology; validation; visualization; writing – review and editing; software; project administration; data curation; supervision; resources. **Sergio Mazzola Poli de Figueiredo:** Resources; supervision; data curation; formal analysis; project administration; writing – review and editing; visualization; validation; methodology; conceptualization; investigation; funding acquisition; writing – original draft. **Fernanda Bellotti Formiga:** Conceptualization; investigation; funding acquisition; writing – original draft; methodology; validation; visualization; writing – review and editing; formal analysis; project administration; data curation; supervision; resources.

## FUNDING INFORMATION

No funding was received for this work.

## CONFLICT OF INTEREST STATEMENT

Dr. Fernanda Bellotti Formiga reports speaker honoraria from Janssen Brazil outside the submitted work. Dr. Sergio Mazzola Poli de Figueiredo reports honoraria from Intuitive and Distal Motion outside the submitted work. All other authors declare no related financial interests or potential conflicts of interest.

## ETHICS STATEMENT

This study is a systematic review and meta‐analysis of previously published studies. Ethical approval was not required.

## Supporting information


Data S1.

## Data Availability

All data used in this review were extracted from published studies. The datasets generated and analysed during the current study are available from the corresponding author upon reasonable request.
